# Supplementary material for: An open-source framework for large-scale, flexible evaluation of biomedical text mining systems
Source: J Biomed Discov Collab. 2008 Jan 29;3:1. doi: 10.1186/1747-5333-3-1 (PMC2276192; doi:10.1186/1747-5333-3-1)
Supplement: Additional file 1 — Evaluation platform source code. The additional file contains the software evaluation framework discussed in this paper. The most current release can be downloaded from SourceForge.net [1]. See the accompanying README file for instructions on installation and use. Also included in the distribution is a collection of UIMA wrappers for some commonly used BioNLP tools and annotated corpora. [file 1747-5333-3-1-S1.zip › bionlp_uima_repository-1.0.1/docs/api/edu/uchsc/ccp/uima/mention/package-tree.html]

edu.uchsc.ccp.uima.mention Class Hierarchy (Center for Computational Pharmacology Util/UIMA API)


|  |  |  |
| --- | --- | --- |
| **Overview** \| **Package** \| Class \| **Tree** \| **Deprecated** \| **Index** \| **Help** | |  |
| **PREV**   **NEXT** | **FRAMES**    **NO FRAMES**     **All Classes** |  |


---


## Hierarchy For Package edu.uchsc.ccp.uima.mention

**Package Hierarchies:**: All Packages

---

## Class Hierarchy

- java.lang.**Object**
  - org.apache.uima.cas.impl.FeatureStructureImpl (implements java.lang.Cloneable, org.apache.uima.cas.FeatureStructure)
    - org.apache.uima.jcas.cas.TOP
      - edu.uchsc.ccp.uima.mention.**CCPMention**
        - edu.uchsc.ccp.uima.mention.**CCPClassMention**- edu.uchsc.ccp.uima.mention.**CCPSlotMention**
            - edu.uchsc.ccp.uima.mention.**CCPComplexSlotMention**- edu.uchsc.ccp.uima.mention.**CCPNonComplexSlotMention**
    - org.apache.uima.jcas.cas.TOP\_Type
      - edu.uchsc.ccp.uima.mention.**CCPMention\_Type**
        - edu.uchsc.ccp.uima.mention.**CCPClassMention\_Type**- edu.uchsc.ccp.uima.mention.**CCPSlotMention\_Type**
            - edu.uchsc.ccp.uima.mention.**CCPComplexSlotMention\_Type**- edu.uchsc.ccp.uima.mention.**CCPNonComplexSlotMention\_Type**

---


|  |  |  |
| --- | --- | --- |
| **Overview** \| **Package** \| Class \| **Tree** \| **Deprecated** \| **Index** \| **Help** | |  |
| **PREV**   **NEXT** | **FRAMES**    **NO FRAMES**     **All Classes** |  |


---
